# *Salmonella* pathogenicity island-14 is a critical virulence factor responsible for systemic infection in chickens caused by *Salmonella gallinarum*

**DOI:** 10.3389/fvets.2024.1401392

**Published:** 2024-05-23

**Authors:** Zuo Hu, Shinjiro Ojima, Zhihao Zhu, Xiaoying Yu, Makoto Sugiyama, Takeshi Haneda, Masashi Okamura, Hisaya K. Ono, Dong-Liang Hu

**Affiliations:** ^1^Department of Zoonoses, Kitasato University School of Veterinary Medicine, Towada, Japan; ^2^Research Center for Drug and Vaccine Development, National Institute of Infectious Diseases, Tokyo, Japan; ^3^College of Veterinary Medicine, Southwest University, Chongqing, China; ^4^Laboratory of Veterinary Anatomy, Kitasato University School of Veterinary Medicine, Towada, Japan; ^5^Laboratory of Microbiology, Kitasato University School of Pharmacy, Tokyo, Japan; ^6^Section of Applied Veterinary Sciences, Division of Veterinary Sciences, Department of Veterinary Medicine, Obihiro University of Agriculture and Veterinary Medicine, Obihiro, Japan

**Keywords:** *Salmonella gallinarum*, fowl typhoid, chicken, SPI-14, systemic infection

## Abstract

*Salmonella enterica* serovar Gallinarum (*S. gallinarum*) is an important host-specific pathogen that causes fowl typhoid, a severe systemic, septicemic, and fatal infection, in chickens. *S. gallinarum* causes high morbidity and mortality in chickens and poses a significant burden and economic losses to the poultry industry in many developing countries. However, the virulence factors and mechanisms of *S. gallinarum*-induced systemic infection in chickens remain poorly understood. In this study, we constructed a *Salmonella* pathogenicity island-14 (SPI-14) mutant strain (mSPI-14) of *S. gallinarum* and evaluated the pathogenicity of mSPI-14 in the chicken systemic infection model. The mSPI-14 exhibited the same level of bacterial growth and morphological characteristics but significantly reduced resistance to bile acids compared with the wild-type (WT) strain *in vitro*. The virulence of mSPI-14 was significantly attenuated in the chicken oral infection model *in vivo*. Chickens infected with WT showed typical clinical symptoms of fowl typhoid, with all birds succumbing to the infection within 6 to 9 days post-inoculation, and substantial increases in bacterial counts and significant pathological changes in the liver and spleen were observed. In contrast, all mSPI-14-infected chickens survived, the bacterial counts in the organs were significantly lower, and no significant pathological changes were observed in the liver and spleen. The expression of interleukin (IL)-1β, IL-12, CXCLi1, tumor necrosis factor (TNF)-α, and interferon (IFN)-γ in the liver of mSPI-14-infected chickens were significantly lower than those in the WT-infected chickens. These results indicate that SPI-14 is a crucial virulence factor in systemic infection of chickens, and avirulent mSPI-14 could be used to develop a new attenuated live vaccine to prevent *S. gallinarum* infection in chickens.

## 1 Introduction

*Salmonella enterica* encompasses a diverse group of serovars, each with broad or specific adaptations to different hosts. Among them, *S. enteritidis* and *S. typhimurium*, which have been widely studied in human and animal infections, are important pathogens with a wide host range that cause self-limiting diarrhea in mammals. *S. gallinarum* is a host-specific serovar that causes severe systemic infectious diseases called fowl typhoid in chickens (1, 2). *S. gallinarum* can invade the liver, spleen, and other organs, causing systemic infections with symptoms such as lethargy and high mortality and causing huge economic losses to the poultry industry, especially in many developing countries (3–5). However, little is known about the virulence factors and mechanisms of *S. gallinarum* inducing systemic infections in chickens (6, 7). It is crucial and necessary to investigate the virulence factors of *S. gallinarum* and the mechanism of pathogen–host interactions to develop effective control measures and prevent the occurrence and spread of fowl typhoid in chickens.

*Salmonella* pathogenicity islands (SPIs) are specific genetic regions found in the genome of bacteria belonging to the *Salmonella* genus. Previous studies have demonstrated that there are multiple SPIs identified in different *Salmonella* serotypes, and each SPI is associated with specific functions related to the pathogenic process, especially playing a crucial role in the ability to cause gastrointestinal infectious diseases (8, 9). SPI contains gene clusters that typically encode virulence factors such as invading secretion systems and elements contributing to bacteria’s ability to evade the host immune system and survive within host cells (7, 9). Different *Salmonella* serovars may possess distinct SPIs that reflect their adaptation to specific hosts or environmental niches. *S. enteritidis* and *S. typhimurium* have a wide host range and can survive in the acidic environment of the stomach (6, 7, 10). After the bacteria reach the intestinal tract, they enter the intestinal cells via the type III secretion system (T3SS) encoded by SPI-1 and subsequently invade intestinal cells, causing intestinal inflammation and promoting the growth of bacteria in the intestine (11–13). Recent studies have reported that SPI-14 encodes a novel regulatory factor called low oxygen-induced factor A (LoiA). When *S. typhimurium* is exposed to a hypoxic environment in the intestinal tract, LoiA binds directly to the promoter and activates the transcription of *hil*D, which further leads to the activation of the master activator *hil*A, suggesting SPI-14 could be a regulator of SPI-1 that is an important virulence regulatory factor in gastrointestinal infections caused by *S. typhimurium* and *S. enteritidis* (7, 14–16).

Although the functions of SPIs in a wide range of host *Enterobacteriaceae* and their roles in mammalian gastroenteritis have been well-studied, the pathogenicity of SPIs in host-specific *S*. Gallimarum, which causes systemic infections in chickens, remains unclear. *S. gallinarum* does not cause significant inflammation in the intestinal tract of chickens but is more likely to cause severe systemic infectious diseases than other *Salmonella* serotypes with broader host ranges (2, 17). Recently, molecular and genetic epidemiological analyses demonstrated that genes of SPI-13, SPI-14, and macrophage-inducible gene *mig*-14 were specifically upregulated in the bird-specific serovar., *S. gallinarum*, and further studies on the role of these genes in the host-specific infection are highly indicated (6, 7). To shed some light on the pathogenic mechanism and elucidate the virulence factors of *S. gallinarum*-induced systemic infection in chickens, in this study, we created a mutant strain, mSPI-14, with deleted SG0835 and SG0836 genes which are encoded in the SPI-14 of *S. gallinarum*, and studied the biological function and pathogenic roles of SPI-14 in chicken systemic infection using our recently established chicken oral inoculation model (5). Our results demonstrate for the first time that the mSPI-14 mutant has reduced resistance to bile salts *in vitro,* and the virulence was significantly attenuated in orally infected chickens *in vivo*, indicating that SPI-14 is an important virulence factor in systemic infection of chickens, and the resulting mSPI-14 can be used as an attenuated live vaccine strain to prevent fowl typhoid caused by *S. gallinarum*.

## 2 Materials and methods

### 2.1 Bacterial strains, plasmids, and culture conditions

*Salmonella enterica* serovar Gallinarum biovar Gallinarum (*S. gallinarum*) NCTC13346 (SG287/91) strain was used as the parent strain (WT) of the gene-deficient strain. pCP20, pKD3, and pKD46 were used to create a strain lacking the SPI-14 genes (SG0835-0836). pCP20 is a temperature-sensitive plasmid, and the genetic recombination enzyme encoded by pCP20 recognizes the Flippase Recognition Target (FRT) sequences located at both ends of the chloramphenicol resistance cassette of pKD3 and removes the chloramphenicol resistance cassette that has replaced the target gene (13). The WT and mSPI-14 were streaked onto Luria-Bertani (LB) agar (Eiken Chemical, Tokyo, Japan) plates using a platinum loop, cultured overnight at 37°C, and the resulting single colony was inoculated into LB broth (Eiken Chemical) and cultured at 37°C with shaking at 150 rpm to prepare a bacterial suspension for next experiments.

### 2.2 Construction of SPI-14 mutant strain (mSPI-14) of *Salmonella gallinarum*

**Figure 1:**
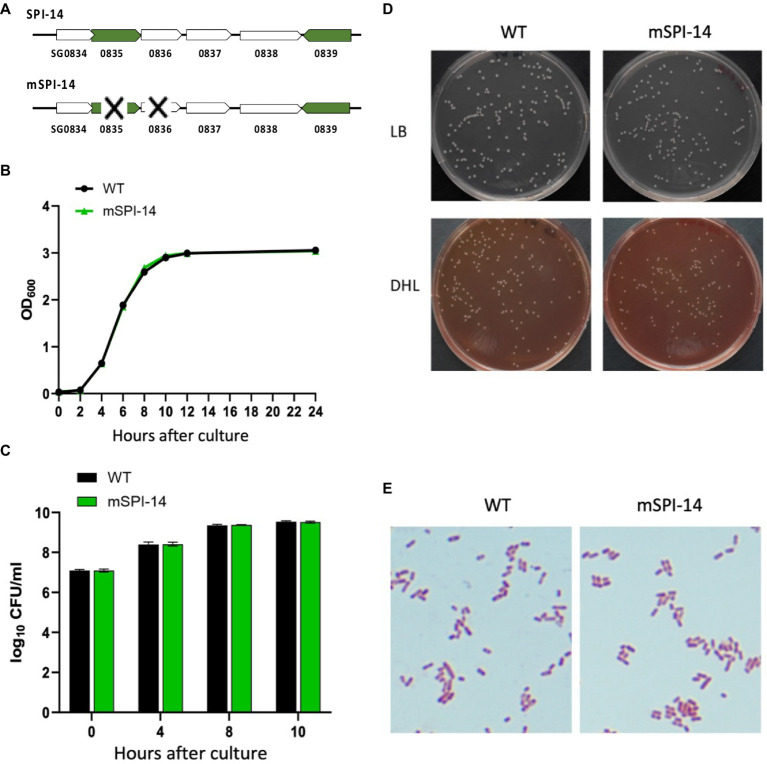
Characteristics of *Salmonella gallinarum* (WT) and mSPI-14. **(A)** Gene structure of SPI-14 and mSPI-14. **(B)** Growth curves of WT and mSPI-14 in LB broth. The bacteria grown in LB broth for 14 h were diluted to OD_600_ = 1.5, inoculated 1/100 into the LB broth, and cultured at 37°C with shaking (150 rpm). The optical density (OD_600 nm_) was measured at 0, 2, 4, 6, 8, 10, 12, and 24 h after inoculation. **(C)** Bacterial counts of WT and mSPI-14 in LB broth. The bacteria were cultivated as described above, and 100 μL of each culture at the indicated time points was serially diluted in LB broth and spread on an LB agar plate. After incubating overnight at 37°C, the colonies on the plate were counted as colony-forming units (CFU). **(D)** Morphology of colonies generated on LB and DHL agar plate medium. **(E)** Morphological and staining characteristics of Gram-stained bacteria.

SPI-14 contains the genes of SG0834-0839, among which 0835 and 0836–0838 are genes with distinct functions (Figure 1A). Therefore, the SG0835 and SG0836 genes contained in SPI-14 were deleted from the parental WT strain according to the previously reported methods (18, 19). In brief, the PCR primers, SG0835-0836F-1 (TATTGAATTATCAGATGCTCCATTCAAATGAGAGACGA GAGTAGGCTGGAGCTGCTTCTT) and SG0835-0836R-1 (GTTATGTTCAGCAATAACTAAA GAAGCCATATTTTCCTCCCATATGAATATCCTCCTTAG), were used to amplify the chloramphenicol resistance cassette, and the SG0835-0836 region was replaced by homologous recombination. To remove the chloramphenicol resistance cassette (Cm) that replaced SG0835-0836 on the genome, pCP20 was transformed into the competent cells of the SG0835-0836::Cm strain in perforated cuvette (0.1 cm gap) using Gene Pulser® II and electroporation (1.8 kV, 25 μF, 200 Ω). The transformed colonies were spread on LB agar plates and cultured at 42°C overnight to remove Cm and shed pCP20 to obtain SGΔ0835-0836. Cm removal was confirmed by colony PCR using the primers SG0835-0836F-2 (TAGCATCGGTCATCAGGCACAAG) and SG0835-0836R-2 (TTTTCACCA TGGGCAAATAT), which anneal to the flanking regions 500 bp away from SG0835-0836. The constructed SGΔ0835-0836 strain was named mSPI-14 mutant.

### 2.3 Comparison of growth properties of WT and mSPI-14 *in vitro*

To compare the growth characteristics of WT and mSPI-14 *in vitro*, each strain was cultured in LB broth at 37°C with shaking at 150 rpm for 14 h. The cultured bacterial culture was diluted with LB broth to an optical density (OD_600 nm_) =1.5, and 100 μL of the diluted suspension was inoculated into 10 mL of LB broth, cultured at 37°C with shaking 150 rpm, and the absorbance (OD_600 nm_) of the culture suspensions were measured at 2, 4, 6, 8, 10, 12, and 24 h after incubation. On the other hand, the number of bacteria in the culture suspensions was counted by applying bacterial culture on LB agar and DHL agar plate, which were incubated at 37°C overnight. Colonies on the plates were counted as colony-forming units (CFU). To compare the colony morphology and cell shape of the WT and mSPI-14, we spread WT and mSPI-14 bacterial liquids on LB agar plates, incubated at 37°C for 18 to 24 h, and observed the colony morphology. Gram stain was performed on fresh single colony smears of each strain, and the shape of the cells was observed under a microscope. To investigate whether growth ability changes between WT and mSPI-14 in different culture conditions, we observed bacterial growth in LB broth containing bile acids (final concentration was 0.01 or 0.02 mM, Sigma-Aldrich), hydrogen peroxide (final concentration of 0.5 or 1.0 mM, Wako, Osaka, Japan), or nalidixic acid (final concentration of 1.25 or 2.5 μg/mL, Wako) in 96-well flat-bottom plates (Greiner BioOne, Kremsmünster, Austria), respectively (19). The plates were incubated at 37°C without shaking, and the bacterial growth at the indicated time points was determined by monitoring the optical density (OD_600 nm_) using a 96-well plate reader (Bio-Rad, model 680 microplate reader, Hercules, CA, United States).

### 2.4 Chickens and oral experiment with infection

Chicken infection experiments were conducted in accordance with the regulations for animal experiments specified in the Animal Welfare Act and the Guide for the Care and Use of Laboratory Animals. The animal experiment protocol was reviewed and approved by the Kitasato University Animal Care Committee (approval numbers: 20–055 and 21–039). Boris brown hens (20-day-old) were housed and provided water and food *ad libitum*. To confirm the absence of *Salmonella* in the flock, fecal swabs were collected from breeding cages, and the bacteriological detection of *Salmonella* was performed before the experimental infection (5, 20). Each chicken was orally inoculated with 10^8^ CFU WT or mSPI-14 strain in a volume of 1.0 mL by oral gavage (the number of chickens in each group is 5). The chickens were housed and kept for 14 days, and any clinical signs were observed and recorded twice a day for monitoring. The above experiment was repeated twice. Every effort was made to reduce animal suffering during experiments. These infection experiments were performed at the same time using the WT and mSPI-14 strains, as well as two other mutant strains (*wecB*::Cm and *trxB*::Cm) that were not shown in this article.

### 2.5 Isolation and enumeration of *Salmonella* in chicken organs after infection

Chickens were inoculated orally with 10^8^ CFU of WT or mSPI-14 or with 1.0 mL of PBS as an uninfected control using the method described above. The chickens were euthanized on days 1, 3, 5, and 7 post-infection. Liver and spleen samples were collected aseptically from 3 to 5 chickens in each group at each time point. The organ samples were then diluted 10-fold with PBS to make homogenates, serially diluted 10-fold with PBS, and plated onto LB agar plates. After incubation at 37°C for 24 h, the numbers of colonies on the plates were determined and calculated as CFU/g organ.

### 2.6 Clinical changes and histopathological examination

Clinical changes, including redness and discoloration of the comb and ruffled feathers, of chickens inoculated with WT, mSPI-14, or PBS were observed, recorded, and evaluated for the occurrence of systemic infection. The extent of inflammation of the liver and spleen from 3 to 4 chickens in each group was investigated at 1, 3, 5, and 7 days after inoculation by observing swelling, congestion, bleeding, redness, and discoloration of the tissues. To assess histological changes and inflammation levels, livers and spleens from each group were fixed in 4% paraformaldehyde (pH 7.4) at 4°C for 24 h and embedded in paraffin. Sections were cut at three levels to a thickness of 4 μm and stained with hematoxylin–eosin (HE) staining. Histological changes, such as the infiltration of inflammatory cells and tissue damage, were recorded and photographed.

### 2.7 Cytokine and chemokine expression

RNA was extracted from the livers of chickens infected with WT or mSPI-14 strain and PBS control on days 3 and 5 after infection, and the expression levels of inflammatory cytokines and chemokines were measured by real-time PCR according to our previously reported methods (17, 19). GAPDH was used as an endogenous control, and IL-1β, IL-6, TNF-α, IL-12, IFN-γ, and CXCLi1 were used as target genes. The expression of the genes was determined using the threshold cycle (ct) value relative to that of the housekeeping gene GAPDH, and the results were expressed as fold changes in the corrected target gene in the infected chickens relative to the uninfected controls.

### 2.8 Statistical analysis

Bacterial counts were expressed in logarithms, and significant differences in the bacterial counts or OD values between WT and mSPI-14 average value at each time point were tested using Student’s *t-*test. In the analysis of cytokine expression levels, significant differences among the three groups in the expression levels of the WT inoculated group, mSPI-14 inoculated group, and uninfected (PBS) control group at each sampling day after inoculation were tested through one-way ANOVA analysis followed by Tukey’s multiple comparison test. Statistical analysis was performed using GraphPad Prism 9.3.0 (GraphPad Software, San Diego, CA, United States), and a significant difference was determined when the *p* values were < 0.05.

## 3 Results

### 3.1 Characteristics of *SPI-14*-deficient *Salmonella gallinarum* (mSPI-14)

We first compared the bacterial growth characteristics of WT and mSPI-14 strains. The bacterial culture with the OD_600_ value of WT or mSPI-14 strain was inoculated into LB liquid medium and cultured with shaking at 37°C for 24 h; no significant difference was observed in the OD_600_ value and the numbers of viable bacteria between the WT and mSPI-14 strains (Figures 1B,C). There was no significant difference in colony size and morphology between the WT and mSPI-14 strains when observing the colonies generated on LB agar and DHL agar plate medium (Figure 1D). We also compared the shapes of Gram-stained bacterial bodies; no abnormality was observed in the shape of the mSPI-14 strain, and no significant differences in morphology were observed between the WT and mSPI-14 strains (Figure 1E).

### 3.2 Resistance of WT and mSPI-14 to bile acids and hydrogen peroxide

**Figure 2:**
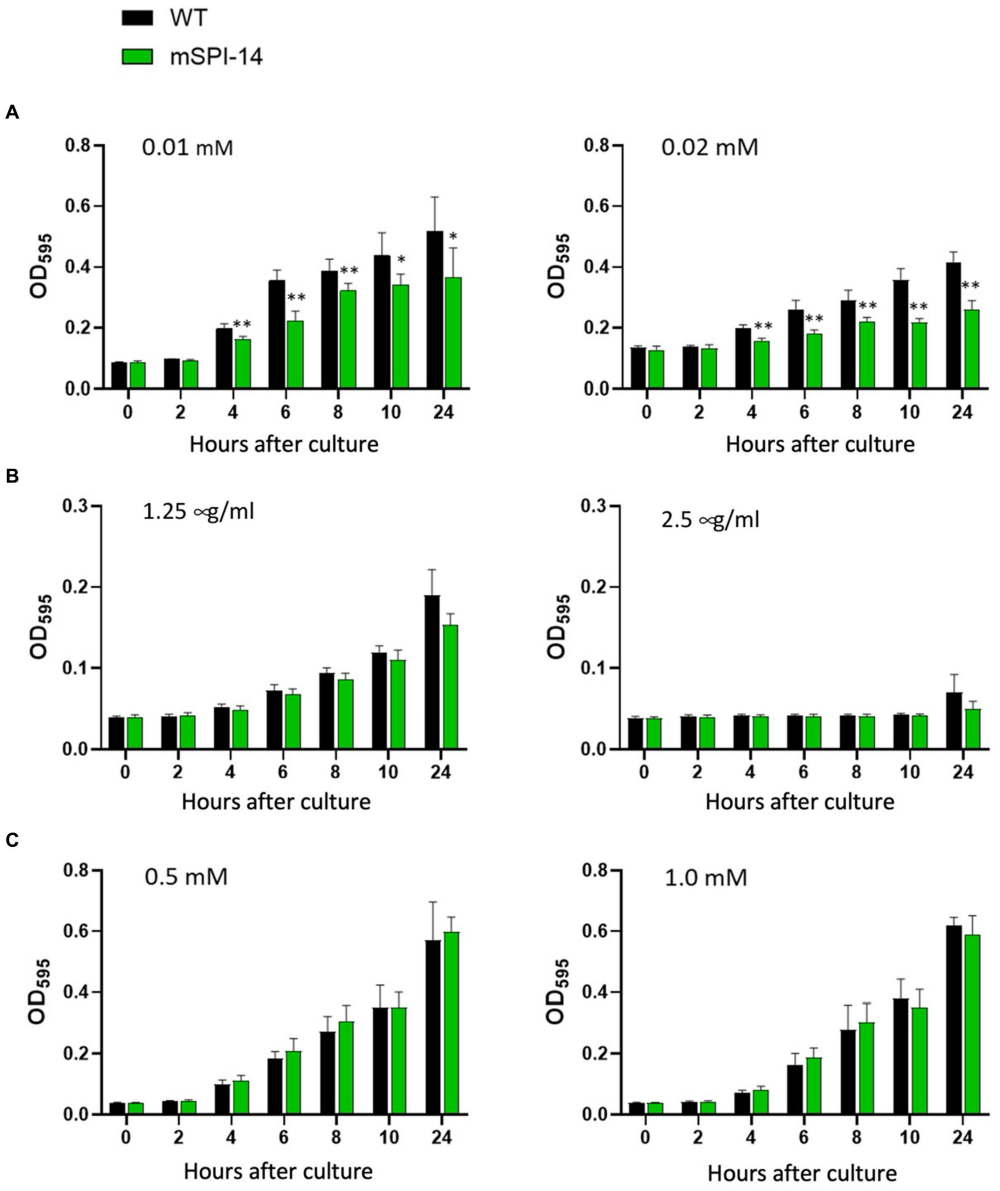
Comparison of bacterial growth characteristics between mSPI-14 and WT in LB broth containing bile acid **(A)**, nalidixic acid **(B)**, or hydrogen peroxide **(C)**. The bacteria were grown in LB broth for 14 h and then diluted to 2.0 × 10^7^ CFU/mL. The diluted bacterial culture was mixed with each reagent (50:50) in 96-well flat-bottom plates and incubated at 37°C without shaking. The optical density (OD_600 nm_) was measured at 0, 2, 4, 6, 8, 10, and 24 h after inoculation. The data are means ± standard deviations based on five wells per group at each time point. The significant difference was shown as *p < 0.05, **p < 0.01.

Since it is known that the wild type of *S. gallinarum* is resistant to bile acids and sensitive to hydrogen peroxide (H_2_O_2_) and nalidixic acid, we evaluated the effects of SPI-14 gene deletion on the resistance to bile acids and sensitivity to H_2_O_2_ and nalidixic acid. In the LB broth with different concentrations of bile acid, the mSPI-14 mutant showed significantly reduced OD values from 4 h after exposure to the concentrations of 0.01 or 0.02 mM bile acid, indicating mSPI-1 is more sensitive to bile acid than the WT strain (*p* < 0.01, Figure 2A). To examine if the SPI-14 is also relevant for antibiotic resistance, the growth of the mSPI-14 and WT strains were analyzed in LB broth supplemented with different concentrations of nalidixic acid. There were similar OD values at 1.25 μg/mL nalidixic acid and no significant difference in the growth between mSPI-14 and WT strains. In addition, both mSPI-14 and WT strains abrogated growth when the concentration of 2.5 μg/mL was added to the media (Figure 2B). In addition, the mSPI-14 showed comparable growth to the WT strain after exposure to LB broth with 0.5 and 1.0 mM H_2_O_2_ (Figure 2C).

### 3.3 Pathogenic role of SPI-14 in an infected chicken *in vivo*

**Figure 3:**
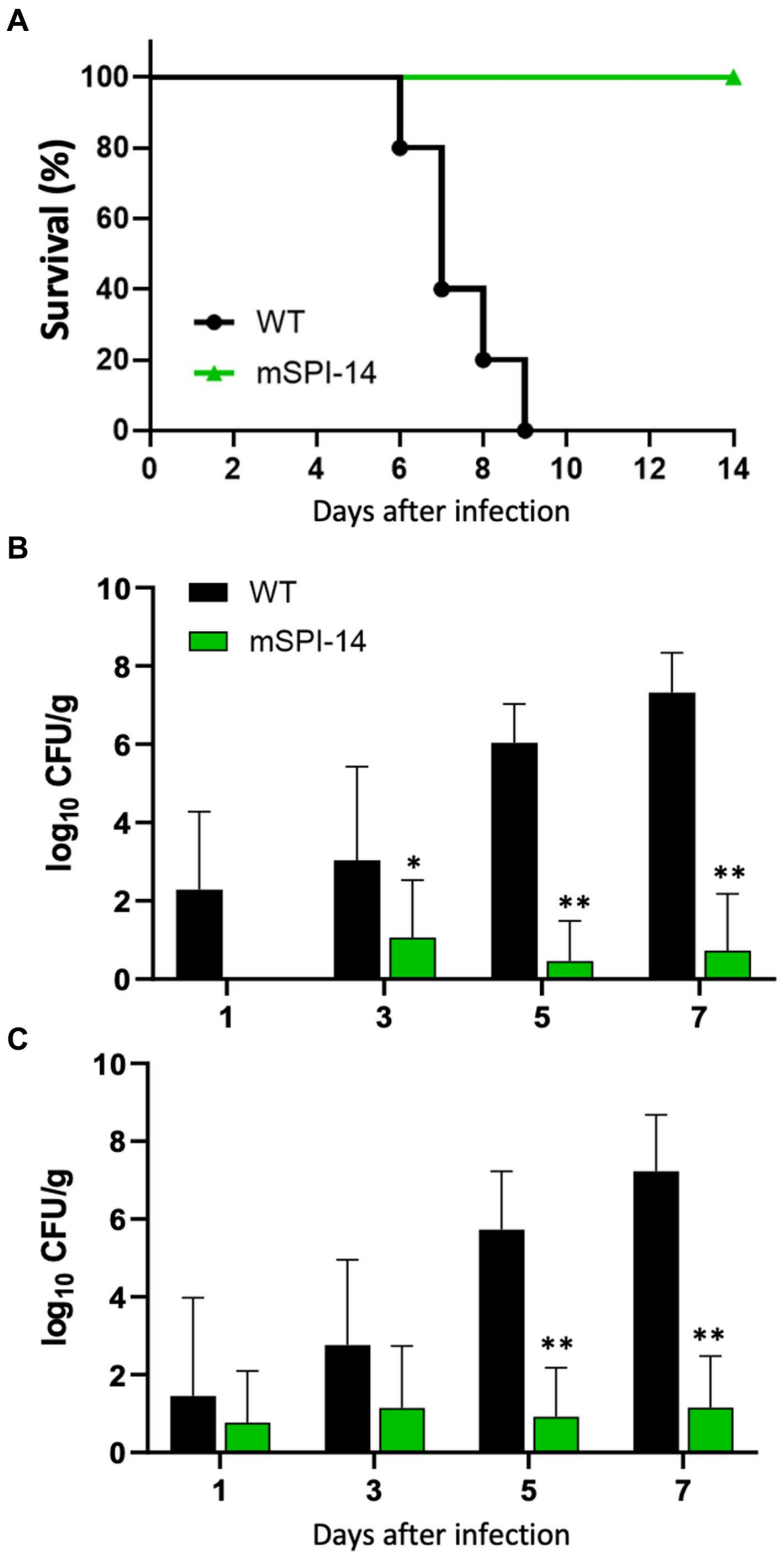
Pathological findings in the chickens orally infected with WT and mSPI-14. **(A)** Survival curve of chickens orally inoculated with WT or mSPI-14. Chickens were orally inoculated with 10^8^ CFU of WT or mSPI-14, and survival of the chickens was recorded for 14 days post-infection. **(B,C)** Viable bacterial counts in the organs of chickens orally inoculated with WT or mSPI-14. Chickens were orally inoculated with 10^8^ CFU of WT or mSPI-14. The bacteria in the liver **(B)** and spleen **(C)** were determined on the 1, 3, 5, and 7 days post-infection. The data are means ± standard deviations based on three to five chickens per group at each time point. The significant differences were shown as *p < 0.05, **p < 0.01.

To investigate the pathogenic roles of SPI-14 in the systemic infection of chickens induced by *S. gallinarum in vivo*, we analyzed the clinical changes and mortality in chickens orally infected with the mSPI-14 or WT strain. The chickens infected with 10^8^ CFU of WT showed significant disturbance or depression, which are the typical clinical symptoms of fowl typhoid, and all of the WT-infected chickens died by 9 days post-infection (Figure 3A). In contrast, the chickens infected with mSPI-14 at the same dose as the WT strain exhibited no significant clinical changes, and all of the chickens survived until the end of the observation period.

### 3.4 Bacterial colonization in the infected chickens

To analyze the spread and colonization of WT and mSPI-14 in the infected chickens, we counted and compared the numbers of viable bacteria in the organs of the infected chickens. The bacterial burdens in the liver and spleen of WT-infected chickens were detected 1 to 7 days after oral infection. The number of viable bacteria in the liver of WT-inoculated chickens continued to increase, and numbers of 1.96 × 10^2^, 1.08 × 10^3^, 1.09 × 10^6^, and 2.09 × 10^7^ CFU/g were detected on days 1, 3, 5, and 7 post-inoculation, respectively (Figure 3B). On the other hand, the number of bacteria in the livers of mSPI-14-infected chickens was 1.15 × 10 CFU/g, 0.29 × 10 CFU/g, and 0.53 × 10 on days 3, 5, and 7 after inoculation, which was significantly lower than that of WT-infected chickens. Similar to the liver, the number of bacteria in the spleen of WT inoculated chickens significantly increased and were detected as 2.85 × 10, 5.75 × 10^2^, 5.41 × 10^5^, 1.7 × 10^7^ CFU/g from day 1 to 7 after inoculation (Figure 3C). The counts of bacteria in the spleen of mSPI-14-infected chickens were also significantly lower than those of the WT-infected chickens (*p* < 0.01).

### 3.5 Macroscopic findings and pathological changes in the infected chickens

**Figure 4:**
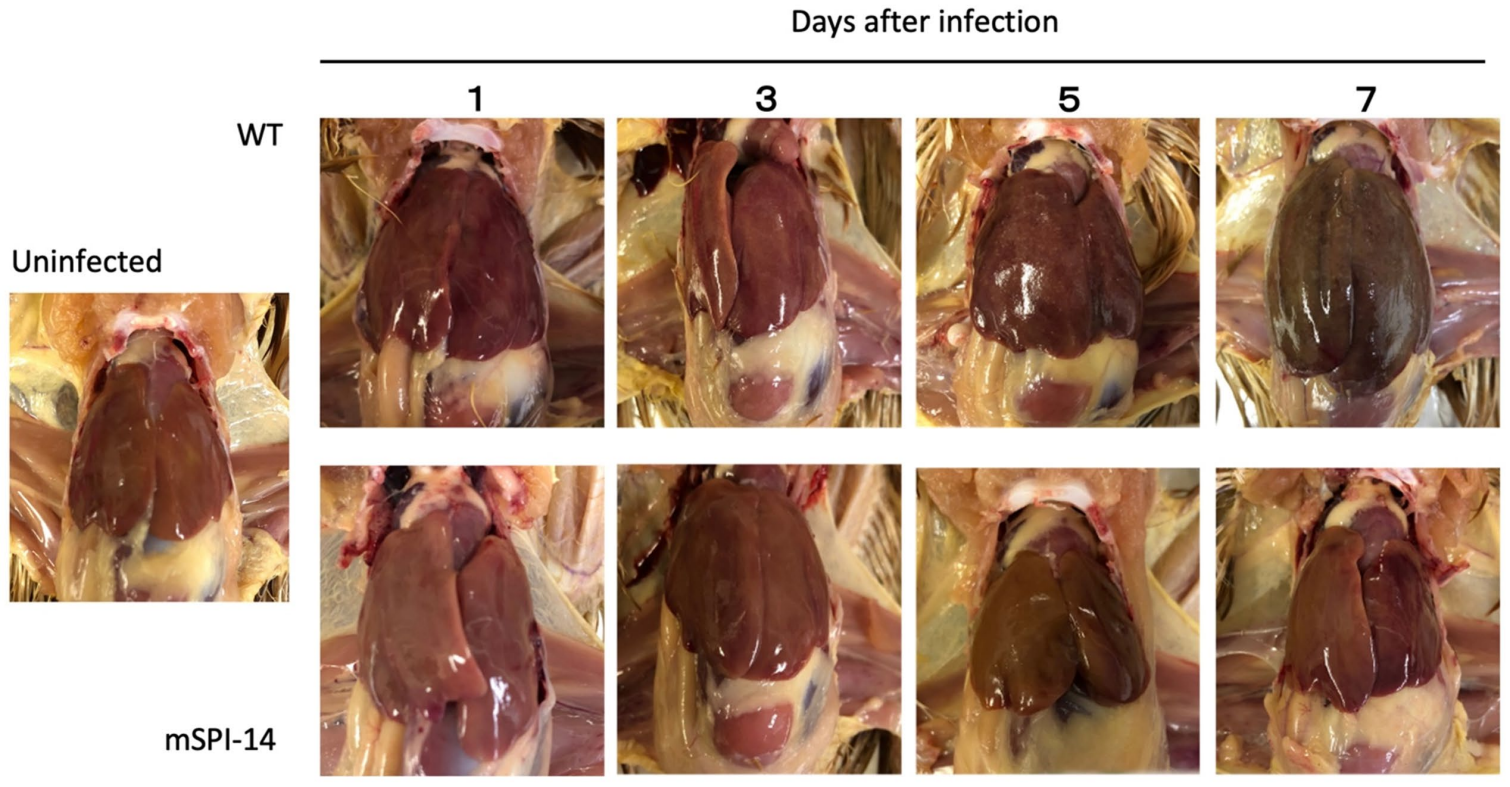
Gross lesions in the liver of chickens orally inoculated with WT and mSPI-14. The livers of pathological changes were observed on 1, 3, 5, and 7 days post-infection. White lesions and small necrotic foci were observed in WT-infected chickens 3 days after infection, and liver hypertrophy and congestion were observed 7 days after infection. Although slight white lesions and small necrotic foci were observed in the chickens infected with mSPI-14 5 days post-infection, no hypertrophy or swelling was observed.

It is known that the most characteristic pathological findings of fowl typhoid are hypertrophy, white lesions, and small necrotic foci, which were observed in the liver of infected chickens (5). We next compared the pathological changes in the liver of chickens infected with WT and mSPI-14 strains. Macroscopic observation of the liver of chickens inoculated with WT showed that white dot-like lesions and small necrotic lesions appeared 5 days after inoculation, and 7 days after inoculation, more white lesions, congestion, turbidity, and swelling appeared. In contrast, no obvious lesions were observed in chickens infected with mSPI-14 (Figure 4).

### 3.6 Histological changes in the infected chickens

**Figure 5:**
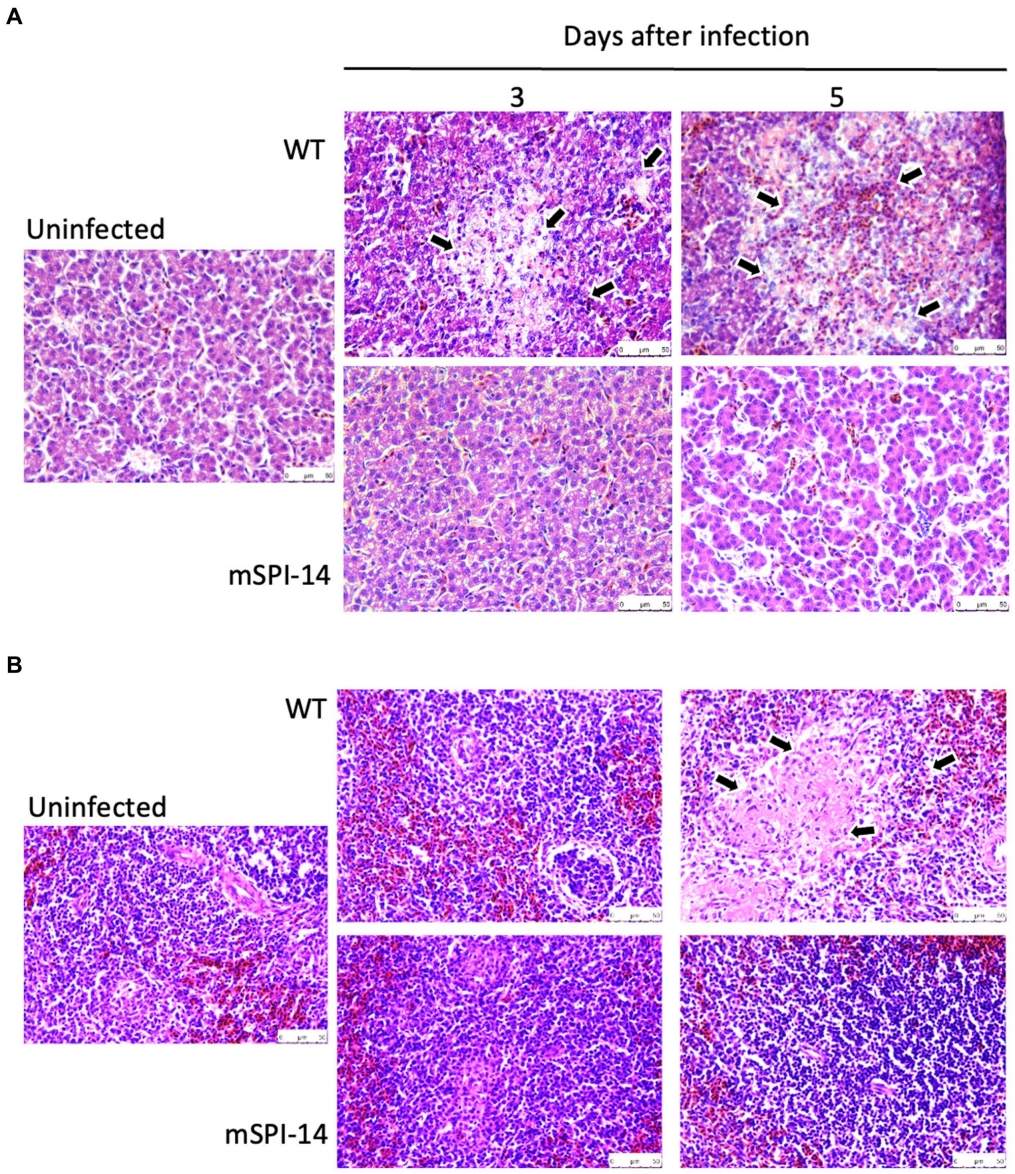
Histopathological changes and microscopic lesions in the livers and spleens of chickens orally infected with WT and mSPI-14. Chickens were orally inoculated with 10^8^ CFU of WT, mSPI-14, or PBS as uninfected controls, and the organs were collected at 3 and 5 days post-infection. Paraffin sections of the organs were prepared and stained with hematoxylin–eosin, magnification×400. **(A)** Liver, the arrows show lesions that were characterized by marked infiltration of heterophils and lymphocytes with degeneration and necrosis. **(B)** Spleen, the arrows show degeneration and necrosis in the white pulp.

Next, we performed the histopathological examination of the liver and spleen of infected chickens to determine whether SPI-14 is related to tissue inflammation caused by *S. gallinarum*. In the liver tissues of WT-inoculated chickens, inflammatory features, such as focal necrosis of hepatocytes, pseudo-eosinophils, and lymphocyte clusters, were observed from days 3 to 5 after inoculation. The extent of inflammation and tissue destruction increased and expanded (Figure 5A). Furthermore, extensive coagulation necrosis was observed in the spleen tissues of WT-inoculated chickens 5 days after inoculation. In contrast, no remarkable histopathological changes were observed in the liver of chickens inoculated with mSPI-14. Furthermore, extensive coagulation necrosis was observed in the spleen tissues of WT-inoculated chickens 5 days after inoculation. There were no significant histopathological changes in the spleen of mSPI-14-inoculated chickens (Figure 5B).

### 3.7 Cytokine and chemokine expression in the infected chickens

**Figure 6:**
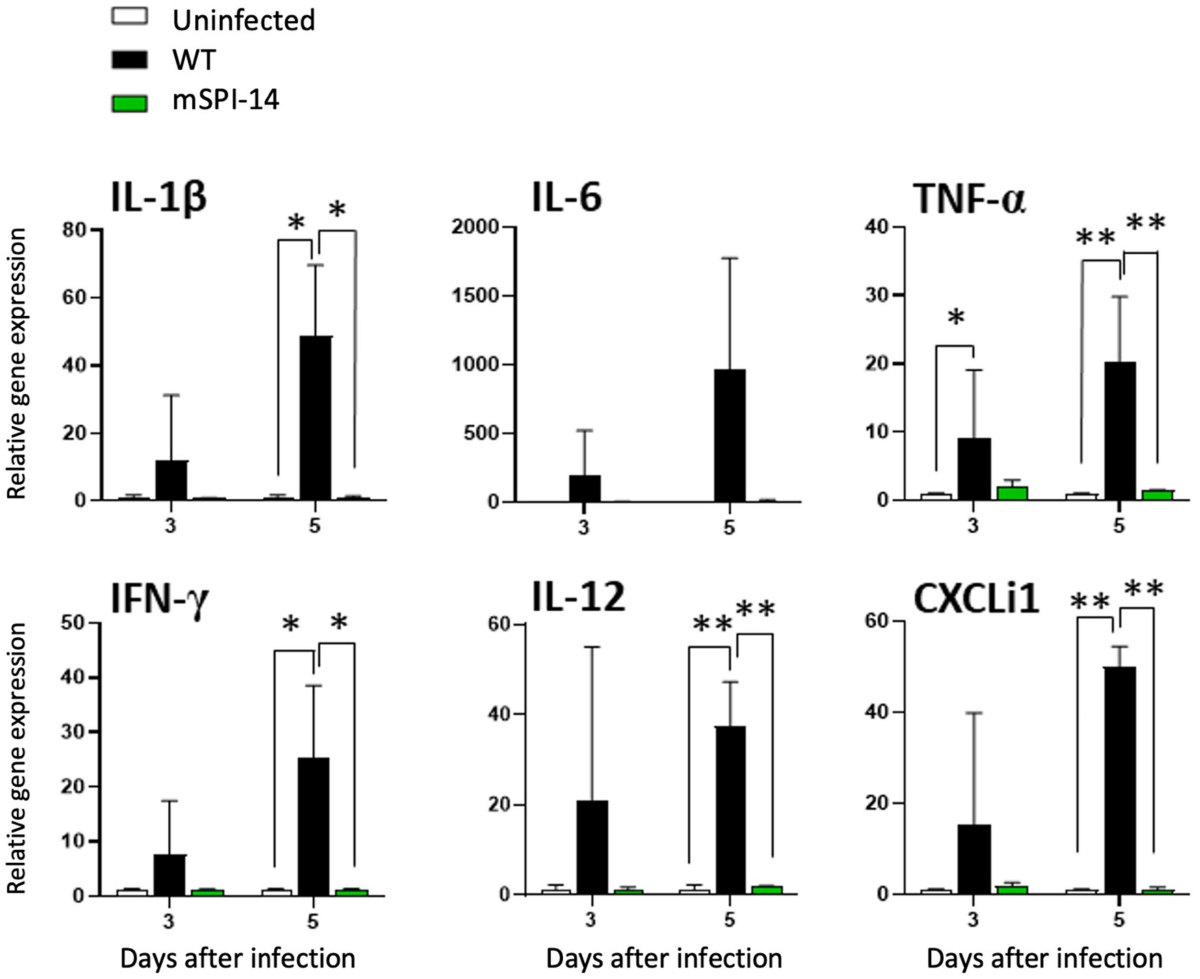
Expression of cytokine and chemokine in the liver of chickens infected with WT and mSPI-14. Chickens were inoculated orally with 10^8^ CFU of WT or mSPI-14. The liver of the chickens was collected on 3 and 5 days post-infection, and the expression of IL-1β, IL-6, TNF-α, IFN-γ, IL-12, and CXCLi1 were determined by quantitative RT-PCR. Data were expressed as means ± standard deviations of fold-changes in gene expression of the organs from infected groups relative to those from the uninfected control group. Statistical analysis was performed using one-way ANOVA analysis followed by Tukey’s multiple comparison test to compare infected chickens with uninfected controls. The significant differences were shown as *p < 0.05, **p < 0.01.

We further analyzed and compared the immune responses of chickens infected with WT and mSPI-14. The expression of cytokine and chemokine genes, IL-1β, IL-6, TNF-α, IFN-γ, IL-12, and CXCLi1 in the liver of chickens were detected 3 to 5 days post-infection. In the livers of the WT inoculated group, the expression level of TNF-α mRNA was increased 3 days after inoculation compared to the uninfected control group (*p* < 0.05, Figure 6). At 5 days after inoculation, the mRNA expression levels of TNF-α, IL-12, and CXCLi1 were more significantly increased (*p* < 0.01), and the increased expression of IL-1β and IFN-γ was also observed at 5 days post-infection (*p* < 0.05). In contrast, no significant changes in cytokine expression were observed in the liver of the mSPI-14 inoculated group compared with the uninfected control group (Figure 6). Importantly, the expression levels of IL-1β, TNF-α, IFN-γ, IL-12, and CXCLi1 in the livers of the mSPI-14-infected group were significantly lower compared with that of the WT-infected group (Figure 6).

## 4 Discussion

*Salmonella gallinarum* is the causative agent of fowl typhoid that causes high morbidity and mortality in poultry and is extremely harmful to the poultry industry with frequent outbreaks, especially in developing countries (21–24). Although some information has been gathered from epidemiological and clinical studies, why *S. gallinarum* causing fatal systemic infection in chickens, as well as its virulence factors and special mechanisms behind adaptation to birds, remain unclear. In the present study, we constructed an SPI-14 deficient mutant of *S. gallinarum* and evaluated the pathogenic effects in a chicken oral infection model. Our results showed for the first time that SPI-14 deletion mutants have significantly reduced resistance to bile salts *in vitro* and that the resulting mSPI-14 strains were significantly attenuated in orally infected chickens *in vivo*, indicating that SPI-14 plays a critical pathogenic role in the systemic infection process of chickens caused by *S. gallinarum*.

The functions of SPIs have been mainly studied in gastrointestinal pathogenic bacteria, such as *S. typhimurium* and *S. enteritidis*, that have a wide range of hosts and cause self-limited diarrhea in humans and animals (7, 9, 25). SPIs have important effects on the bacterial physiology and interactions with the environment and gastrointestinal tract of mammals (7, 26). Of them, SPI-14 can activate the transcription of the invasion regulator *hil*D in *S. typhimurium* (7). Recent studies have reported that SPI-14 (STM14-1008) encodes LoiA, which is a novel regulator of SPI-1. LoiA directly binds to the promoter and activates the transcription of *hil*D, subsequently leading to the activation of *hil*A, a master activator of SPI-1 (7, 8, 27). Deletion of *loi*A significantly reduced the transcription of *hil*A, *hil*D, and other representative SPI-1 genes under low O_2_ conditions (7, 28). SPI-14 promotes *S. typhimurium* virulence by affecting invasion, and the *loi*A is a virulence determinant of SPI-14. Deletion of a single *loi*A gene or the entire SPI-14 region severely attenuated the virulence of *S. typhimurium* in mice oral infection experiments (6, 7). Additionally, LoiA can also regulate multiple virulence-related genes in *S. typhimurium* through the negative regulation of Lon protease (29). Recent studies have demonstrated that *S. enteritidis* and *S. typhimurium* may share the same virulence mechanisms for epithelial cell invasion and survival within macrophages (9, 25, 30).

However, compared with the functional studies of SPI-14 in *S. typhimurium*, *S. enteritidis*, and other Gram-negative bacteria, little is known about the function and pathogenic effect of SPI-14 in the bird-specific bacterium, *S. gallinarum*, that causes severe systemic infection rather than self-limited diarrhea in chickens. Our present study showed that the mSPI-14 strain of *S. gallinarum* showed no significant changes in the growth kinetics and colony morphology, but the resistance to bile acid had significantly reduced compared with those of the WT strain *in vitro* (Figure 2). Importantly, the pathogenicity of the mSPI-14 mutant was also significantly attenuated during the oral infection experiments of chickens *in vivo*. mSPI-14 did not cause serious systemic infection in chickens, and the number of viable bacteria in the organs was significantly lower than that of the WT-infected chickens (Figure 3). The mSPI-14-deleted SG0835 and SG0836 genes showed decreased tolerance to bile acids and significantly attenuated pathogenicity in chickens. The relationship between the resistance to bile acids and bacterial virulence in systemic infections should be investigated in more detail in subsequent studies. A previous study reported that bile acids can suppress the expression of genes related to *Salmonella* invasion by inactivating HilD (26). A recent study also demonstrated that genes SG0835-G0839 (except SG0836) of SPI-14 are uniquely regulated in the bird-specific *S. gallinarum*, suggesting that SPI-14 may play different pathogenic roles between *S. gallinarum* serovar and other serovar such as *S. enteritidis* and *S*. Dublin in which the genes were not be regulated (6). Our results and these reports strongly indicate that genes within SPI-14, including the SG0836 gene, are critical virulence genes for chicken systemic infections (6, 26).

To date, studies on the effects of *Salmonella enterica* SPI-14 on systemic infection of chickens *in vivo* are very limited. In our recent studies, the chickens infected with *S. gallinarum* WT died after showing clinical symptoms such as depression and ruffled feathers, and gross examination of the liver showed white punctate lesions and hepatomegaly (5, 19). Furthermore, in histopathological analysis, remarkable and widespread inflammatory reactions were observed in organs, such as focal necrosis, heterophils, lymphocyte clusters in the liver, and coagulative necrosis in the spleen (19). In this study, in contrast, chickens infected with the mSPI-14 mutant showed no significant clinical symptoms, had a 100% survival rate, and showed no differences in gross findings and/or pathological changes compared to the uninfected group. Furthermore, the production of pro-inflammatory cytokines and chemokines in the chickens infected with mSPI-14 was also significantly lower than that in the chickens infected with WT. These results further indicated that the deletion of SPI-14 is involved in the reduced virulence of *S. gallinarum* in systemic infection of chickens. The SPI-14 gene cluster has been identified as crucial for SPI-1 expression. SPI-1 and SPI-2 in both *S. enteritidis* and *S. typhimurium* encode the Type III Secretion System (T3SS), playing a pivotal role in cell invasion and intracellular survival within the host’s intestinal tract (29, 31, 32). Some SPIs are ubiquitous and distributed among all *Salmonella* species, while other SPIs may be associated with specific serotypes, revealing adaptive advantages and host specificity (10, 24). In fact, some *S. gallinarum* strains from clinically infected chickens lack SPI-1, whereas SPI-1 mutant strains of *S. gallinarum* still show their ability to persist within chicken macrophages (29, 33). These studies indicate that SPI-1 may not be necessary for the pathogenicity of *S. gallinarum* in systemic infection of chickens. SPI-14 plays a key role in systemic infection of chickens caused by *S. gallinarum*, possibly due to its different functions and/or pathways from the *S. typhimurium* and *S. enteritidis*, which are mainly causing self-limiting diarrhea in animals. Given the potential of attenuated strains as vaccines for controlling *S. gallinarum* (34, 35), our study demonstrated that the constructed mSPI-14 mutants serving as viable attenuated vaccine strains may have great development prospects.

## Data availability statement

The datasets presented in this study can be found in online repositories. The names of the repository/repositories and accession number(s) can be found in the article/supplementary material.

## Ethics statement

The animal study was approved by the Kitasato University Animal Care Committee. The study was conducted in accordance with the local legislation and institutional requirements. The chicken infection experiments were conducted in accordance with the regulations for animal experiments specified in the Animal Welfare Act and the Guide for the Care and Use of Laboratory Animals. Every effort was made to reduce animal suffering during experiments. The animal experiment protocol was reviewed by the Kitasato University Animal Care Committee and approved by the President of Kitasato University (approval numbers: 20–055 and 21–039).

## Author contributions

ZH: Data curation, Investigation, Methodology, Visualization, Writing – original draft. SO: Data curation, Investigation, Methodology, Visualization, Writing – review & editing. ZZ: Investigation, Writing – original draft. XY: Data curation, Investigation, Visualization, Writing – review & editing. MS: Investigation, Methodology, Writing – review & editing. TH: Writing – review & editing. MO: Methodology, Writing – review & editing. HO: Conceptualization, Investigation, Methodology, Writing – review & editing. D-LH: Conceptualization, Funding acquisition, Methodology, Project administration, Supervision, Visualization, Writing – review & editing.
